# Trends in management and outcomes of COVID patients admitted to a Swiss tertiary care hospital

**DOI:** 10.1038/s41598-023-32954-1

**Published:** 2023-04-12

**Authors:** Christophe Marti, Christophe Gaudet-Blavignac, Jeremy Martin, Christian Lovis, Jérôme Stirnemann, Olivier Grosgurin, Fiona Novotny, Anne Iten, Aline Mendes, Virginie Prendki, Christine Serratrice, Pauline Darbellay Farhoumand, Nour Abidi, Pauline Vetter, Sebastian Carballo, Jean-Luc Reny, Amandine Berner, Angèle Gayet-Ageron

**Affiliations:** 1 Faculty of Medicine, University of Geneva, Geneva, Switzerland; 2 Division of General Internal Medicine, Geneva University Hospitals, Rue Gabrielle Perret-Gentil 4, 1211 Geneva 14, Switzerland; 3 Division of Medical Information Science, Geneva University Hospitals, Geneva, Switzerland; 4 Department of Radiology and Medical Informatics, Faculty of Medicine, University of Geneva, Geneva, Switzerland; 5 Infection Control Program, Geneva University Hospitals, Geneva, Switzerland; 6 Division of Rehabilitation and Geriatrics, Geneva University Hospitals, Geneva, Switzerland; 7 Division of Internal Medicine of the Elderly, Geneva University Hospitals, Geneva, Switzerland; 8 Department of Acute Medicine, Geneva University Hospitals, Geneva, Switzerland; 9 Geneva Center for Emerging Viral Diseases, Geneva University Hospitals, Geneva, Switzerland; 10 Division of Clinical Epidemiology, Geneva University Hospitals, Geneva, Switzerland

**Keywords:** Infectious diseases, Respiratory tract diseases, Outcomes research

## Abstract

Two successive COVID-19 flares occurred in Switzerland in spring and autumn 2020. During these periods, therapeutic strategies have been constantly adapted based on emerging evidence. We aimed to describe these adaptations and evaluate their association with patient outcomes in a cohort of COVID-19 patients admitted to the hospital. Consecutive patients admitted to the Geneva Hospitals during two successive COVID-19 flares were included. Characteristics of patients admitted during these two periods were compared as well as therapeutic management including medications, respiratory support strategies and admission to the ICU and intermediate care unit (IMCU). A mutivariable model was computed to compare outcomes across the two successive waves adjusted for demographic characteristics, co-morbidities and severity at baseline. The main outcome was in-hospital mortality. Secondary outcomes included ICU admission, Intermediate care (IMCU) admission, and length of hospital stay. A total of 2′983 patients were included. Of these, 165 patients (16.3%, n = 1014) died during the first wave and 314 (16.0%, n = 1969) during the second (*p* = 0.819). The proportion of patients admitted to the ICU was lower in second wave compared to first (7.4 vs. 13.9%, *p* < 0.001) but their mortality was increased (33.6% vs. 25.5%, *p* < 0.001). Conversely, a greater proportion of patients was admitted to the IMCU in second wave compared to first (26.6% vs. 22.3%, *p* = 0.011). A third of patients received lopinavir (30.7%) or hydroxychloroquine (33.1%) during the first wave and none during second wave, while corticosteroids were mainly prescribed during second wave (58.1% vs. 9.1%, *p* < 0.001). In the multivariable analysis, a 25% reduction of mortality was observed during the second wave (HR 0.75; 95% confidence interval 0.59 to 0.96). Among deceased patients, 82.3% (78.2% during first wave and 84.4% during second wave) died without beeing admitted to the ICU. The proportion of patients with therapeutic limitations regarding ICU admission increased during the second wave (48.6% vs. 38.7%, *p* < 0.001). Adaptation of therapeutic strategies including corticosteroids therapy and higher admission to the IMCU to receive non-invasive respiratory support was associated with a reduction of hospital mortality in multivariable analysis, ICU admission and LOS during the second wave of COVID-19 despite an increased number of admitted patients. More patients had medical decisions restraining ICU admission during the second wave which may reflect better patient selection or implicit triaging.

## Introduction

On March 11th 2020, the World Health Organization (WHO) officially declared that the coronavirus disease 2019 (COVID-19) was a pandemic after first cases had been observed in Wuhan city in the Hubei province of China in late November 2019, and rapidly spread throughout the world from Eastern to Western countries. In Switzerland, a first flare of SARS-CoV-2 infections occurred in February 2020 and was followed by an unexpectedly greater second wave in October 2020 compelling authorities to restore a partial lockdown^1^.

The cantons of Geneva and Ticino were particularly affected by the sanitary crisis with incidences of COVID-19 up to 10 times higher than other Swiss cantons^2^. In Geneva, all COVID-19 patients requiring hospital admission during the first wave were admitted to the Geneva University Hospital, the only public hospital of the canton. This strategy compelled the Geneva University hospital (HUG) to increase its intensive care unit (ICU), intermediate medical care unit (IMCU) and acute care capacity by stopping elective surgery, non-COVID research, elective medical consultations and transferring non COVID patients to private hospitals of the canton. During this period, therapeutic strategies and hospital organisation have been constantly adapted based on emerging evidence regarding the management of SARS-CoV-2. A multidisciplinary group including internal medicine, infectious diseases, intensive care medicine and pulmonology specialists was created to guide therapeutic approach of COVID-19 and ensure rapid implementation of emerging evidences. A multimodal strategy was used to disseminate these recommendations through a dedicated website^3^ and smartphone applications such as Spectrum (https://spectrum.app) or HeadtoToe (http://www.headtotoegeneva.com ).

In the context of the ongoing pandemic and shortage of resources, therapeutic aspects regarding antiviral or anti-inflammatory therapy have progressively evolved over the two successive COVID waves as well as the strategy regarding the use of non-invasive respiratory support such as High-Flow Nasal Oxygen (HFNO) and Non-invasive Ventilation (NIV)^4^.

Hydroxycloroquine and lopinavir/ritonavir were recommended during the first wave based on in vitro efficacy data^5^, while evidence supporting the administration of dexamethasone in patients suffering from SARS-CoV-2 pneumonia became available in June 2020^6^. In accordance with WHO recommendations, remdesivir was not recommended for hospitalized patients during the study period. Thromboprophylaxis was recommended during the study period using standard prophylactic doses of low molecular-weight heparin (LMWH) (0.5 mg/kg once daily) for acute care patients and “intermediate doses” LMWH (0.5 mg/kg twice daily) for ICU or IMCU patients.

Regarding respiratory support strategies, there was some reluctance at the beginning of the pandemic to provide HFNO or NIV due to potential aerosolization hazards and the fear of delayed intubation^4,7^. However, experience during the first COVID-19 wave in Switzerland and other countries led to a more systematic use of these non-invasive respiratory support, awake prone positioning and better recognition of the need for invasive mechanical ventilation (IMV)^8–12^. Moreover, admission criteria to the IMCU differed between the two periods as patients ineligible to ICU according to their goals of care were admitted to the IMCU during the second wave, but not during first.

The aim of our study was to describe the evolution in the management of COVID-19 inpatients through two first successive COVID waves at a large teaching hospital and the association between these adaptations and patient outcomes.

## Methods

### Study setting

This study is a retrospective, observational, monocentric cohort study conducted at HUG, a primary and tertiary care hospital in Geneva (HUG), Switzerland during the first (February to May 2020) and second waves (September to December 2020) of the pandemic.

### Data source/measurements

All demographic, clinical, biological and outcome data were retrieved from the patient electronic medical records. Data was extracted from a database designed for COVID-19 related data. This database contains all clinical information and general consent information available in HUG for patients tested positive for SARS-COV-2 or flagged as positive or suspect in the electronic health record since the beginning of the pandemic^13^. The study was approved by the institutional ethics committee (BASEC number: 2021–00,302) in accordance with the Federal Human Research Act (art.34)^14^. All participants were informed of their inclusion into this database and given the opportunity to withdraw consent. Patients and Public were not involved in the conduct of the study. The STROBE (Strengthening the Reporting of Observational studies in Epidemiology) statement was followed to ensure rigorous analysis and transparent reporting^15^. Analyses were performed according to a pre-defined protocol.

### Participants

Our cohort included adult patients hospitalized at HUG for acute community-acquired SARS-CoV-2infection for more than 24 h. In order to limit potential selection bias, we included all consecutive patients except those with a documented refusal to the use of their clinical data. The diagnosis of COVID was established by the physician in charge and documented in a dedicated section of the patient electronic medical record. The diagnosis of COVID-19 was based on the presence of a positive RT-PCR test on a nasopharyngeal swab or lower respiratory tract sample performed at our virology laboratory. Alternatively, patients with a positive RT_PCR test performed before hospital admission or with a negative RT-PCR test and suggestive clinical (cough, fever, anosmia) and radiological presentation (bilateral infiltrates on chest radiography or computed tomography) with a documented seroconversion during hospital course were considered as COVID-19 cases. To exclude nosocomial cases, confirmation of diagnosis had to occur within 7 days before and 72 h after admission.

### Outcomes

Main outcomes: The main outcome was in-hospital mortality. In-hospital mortality was defined as living status at discharge based on the medical record. For patients discharged to post-acute care facilities of the Geneva University Hospitals, status at discharge from post-acute care was considered.

Secondary outcomes: Secondary outcomes included ICU admission, hospital length of stay (LOS), IMCU admission and occurrence of pneumothorax. ICU and IMCU admissions were defined as any transfer to the ICU/IMCU occurring during hospital stay for acute COVID-19 infection regardless of the treatment administered or LOS.

### Independent predictive variables

In order to provide a comparison between the two waves demographic variables (age, gender), comorbidities and markers of severity were compared between study periods. Comorbidities were collected according to a modified Charlson Comorbidity Index^16,17^. International Classification of Diseases (ICD) codes were used to retrieve comorbidities of each patient. Markers of severity included vital parameters at admission (pulse rate, systolic blood pressure) markers of respiratory insufficiency (respiratory rate and SpO_2_/FiO_2_ ratio), and biological variables such as inflammation markers (C-reactive protein, D-Dimers) or markers of organ dysfunction (creatinine, blood pH). For these variables, the worst value during the first 24 h after admission was used. For therapeutic limitation instructions (not to be resuscitated (NTBR) or no ICU admission), we used the last prescription documented in the patient electronic record during the COVID-19 related hospital stay.

### Statistical analysis methods

Continuous variables were reported as means (SD) and ranges. Categorical variables were described by frequencies and relative proportions. Between-group comparisons were performed using Chi-2 or Fisher’s exact tests depending on applicability criteria, for categorical variables; for continuous variables, Student t test with Welch’s correction for unequal variances was performed due to large sample sizes.

In order to report the adjusted associations between pre-specified factors and 30-day mortality, we performed competing risk survival analysis for which time-to-event was calculated from the date of the first COVID-19 diagnosis to either the date of outcomes or the censoring date. We accounted for competing risks with 30-day mortality being the outcome of interest; date of discharge at home as competing risks. We performed univariate and multivariate Fine and Gray models to determine the risk factors of 30-day mortality, adjusting for sex (male, female), age (as continuous), COVID-19 wave (first, second), Charlson comorbidity index (0, 1–2, 3–4, ≥ 5), obesity (no, yes), creatinine ≥ 106 µmol/L (no, yes), urea ≥ 7.5 mmol/L (no, yes), SpO_2_/FiO_2_ categories (< 3.70, 3.70–4.50, ≥ 4.50), CRP ≥ 10mgl/L (no, yes), respiratory rate ≥ 20/min, and corticosteroids use (no, yes). These variables were selected by investigators based on availability and clinical relevance in order to take into account potential confounders for between wave comparisons. Associations were reported by hazard ratio (HR) and 95% confidence interval (95% CI). We made a complete case analysis and excluded all observations with missing variables. We did not apply any correction for missingness. Significance level was set at 0.05 for all comparisons. For all continuous variables, we graphically verified the linearity assumption of the hazard ratio for continuous variables; if it was not respected we chose to present categorized variables using clinically relevant cut-offs. Additionally, a post-hoc sensitivity analysis using continuous variables was computed. Post-hoc analyses were performed to compare characteristics of patients treated or not treated with corticosteroids between the two waves using logistic regression models explaining corticosteroids use (yes/no) and interaction terms between each characteristic and the time-period (second vs. first wave), as independent variables to assess the change in the associations between each characteristic and corticosteroids use. All interaction terms that were statistically significant were introduced in a final multivariable logistic regression model to report the adjusted/independent associations between prognostic variables at baseline and corticosteroids use by odds ratios and 95% CI. Statistical analyses were performed using STATA IC 17.0.

### Ethisdsdcs approval and consent to participate

The study was approved by the institutional ethics committee (Commission Cantonale d’Ethique et de Recherche (CCER) BASEC number: 2021–00,302). All the experiments were carried out in accordance with relevant guidelines and regulations with the Federal Human Research Act (art.34) and informed written consent was obtained from the participants^14^.

## Results

### Participants

A total of 2983 patients, 1′014 during the first wave and 1′969 during the second wave were included. 2822/2983 (94.6%) patients had a positive RT-PCR test at our laboratory of virology, while the remaining 161 patients (5.4%) had a diagnosis of COVID-19 infection documented by the physician in charge. Characteristics of included patients are provided in Table 1. Compared to first wave, patients admitted to the hospital during the second wave tended to be older (mean age 71.8 vs. 68.4), more comorbid, had significantly higher values of D-Dimers, higher creatinine, lower pH and a statistically higher respiratory rate, while body mass index (BMI), C-reactive protein and PaO_2_/Fio_2_ ratios did not significantly differ across the two waves.

**Table 1:** Patient characteristics during the two successive COVID waves at HUG.

Variables	Overall (n = 2′983)	First wave (n = 1′014)	Second wave (n = 1′969)	*p* value
Mean age (± SD, range, missing), years	70.6 (± 18.0, 17–104, 0)	68.4 (± 18.2, 18–103, 0)	71.8 (± 17.7, 17–104, 0)	< 0.001*
Male gender, n (%), missing	1′382 (46.3), 0	468 (46.2), 0	914 (46.4), 0	0.890
Female gender, n (%), missing	1′601 (53.7), 0	546 (53.8), 0	1′055 (53.6), 0	
Mean Charlson comorbidity index (± SD, range, missing)	2.08 (± 2.4, 0–15, 351)	2.02 (± 2.5, 0–15, 114)	2.12 (± 2.4, 0–15, 237)	0.329*
Charlson comorbidity index category, n (%)				< 0.001
0	943 (35.8)	367 (40.8)	576 (33.2)	
1–2	761 (28.9)	214 (23.8)	547 (31.6)	
3–4	533 (20.3)	178 (19.8)	355 (20.5)	
≥ 5	395 (15.0)	140 (15.6)	255 (14.7)	
Mean d-dimer (± SD, range, missing) in ng/L	1683.1 (± 1525.8, 191–9761, 2018)	1542.5 (± 1445.5, 220–9298, 885)	1703.8 (± 1537.5, 191–9761, 1133)	0.241*
Mean creatinine (± SD, range, missing) in µmol/L	105.5 (± 86.9, 84, 28–1413, 439)	100.6 (± 78.0, 28–1267, 130)	108.1 (± 91.2, 33–1413, 309)	0.031*
Mean Urea (± SD, range, missing), mmol/L	7.61 (± 5.66, 0.8–60.3, 458)	7.03 (± 5.50, 1.2–60.3, 125)	7.93 (± 5.72, 0.8–54.9, 333)	< 0.001*
Mean pH (± SD, range, missing)	7.44 (± 0.08, 6.99–7.72, 2286)	7.45 (± 0.08, 7.03–7.64, 704)	7.43 (± 0.08, 6.99–7.72, 1582)	< 0.006*
Mean CRP (± SD, range, missing) in mg/L	81.7 (± 78.5, 0.3–634.4, 499)	78.7 (± 72.8, 0.3–402.8, 136)	83.3 (± 81.4, 0.3–634.4, 363)	0.149*
Mean SpO_2_/FIO_2_ (± SD, range)	3.67 (± 0.99, 0.8–4.76, 215)	3.67 (± 0.98, 0.9–4.76, 77)	3.67 (± 1.00, 0.8–4.76, 138)	0.792*
Mean heart rate (± SD, range)	99.5 (± 21.9, 45–248, 23)	98.5 (± 19.5, 56–200, 10)	99.9 (± 23.1, 45–248, 13)	0.086*
Mean systolic BP (± SD, range) in mmHg	103.7 (± 21.4, 3–180, 29)	103.7 (± 20.6, 7–165, 11)	103.7 (± 21.8, 3–180, 18)	0.953*
Mean respiratory rate (± SD, range, missing)	30.2 (± 10.2, 0–105.9, 374)	29.3 (± 8.9, 0–94, 118)	30.7 (± 10.8, 0–105.9, 256	< 0.001
Limitation regarding ICU admission, n (%), missing	1256 (45.2), 204	377 (38.7), 42	879 (48.6), 162	*< 0.01

*Student test for comparisons of continuous variables; **Chi-2 tests for comparisons of categorical variables.

### Treatments received

Treatments received during the two waves differed significantly: lopinavir/ritonavir and hydroxychloroquine were administered to about one third of patients during the first wave and none during second wave; the majority of patients (58.1%) received corticosteroids during the second wave and a few (9.1%) during first. More patients received antibiotics during first wave (75.2%) than during second wave (53%, *p* < 0.001). Interleukine-6 inhibitors were also given at a lesser extent during the second wave compared to first, but overall, to very few patients (Table 2).

**Table 2:** Treatment administered during the two successive waves.

Treatment	Overall (n = 2′983)	First wave (n = 1′014)	Second wave (n = 1′969)	*p* value
Corticosteroids, n (%)				
No	1′747 (58.6)	922 (90.9)	825 (41.9)	< 0.001
Yes	1′236 (41.4)	92 (9.1)	1′144 (58.1)	
Lopinavir/r, n (%)				–
No	703 (69.3)	703 (69.3)	–	
Yes	311 (30.7)	311 (30.7)	–	
Remdesivir, n(%)		− 1014 (100)		–
No	2887 (96.8)	–	1873 (95.1)	
Yes	96 (3.2)		96 (4.9)	
Hydroxychloroquin, n (%)		678 (66.9)	–	
No	678 (66.9)	336 (33.1)		
Y es	336 (33.1)			
Any antibiotics, n (%)				
No	1′177 (39.5)	251 (24.8)	926 (47.0)	< 0.001
Yes	1′806 (60.5)	763 (75.2)	1′043 (53.0)	
Anti-Il6, n (%)				
No Yes	2′975 (99.7) 8 (0.3)	1′008 (99.4) 6 (0.6)	1′967 (99.9) 2 (0.1)	0.022*

### Outcomes

Overall, 479 patients (16.1%, n = 2983) died during hospitalization (Table 3). Crude in-hospital mortality did not differ between the two waves (16.3% vs. 16%; *p* = 0.819). Compared to first wave, patients admitted during the second wave were less likely to be admitted to the ICU (7.4 vs. 13.9%; *p* < 0.001), more likely to be admitted to the IMCU (26.6 vs. 22.3%, *p* = 0.011) and had a reduced overall LOS (35.8 ± 48.1 versus 40.9 ± 75.3 days, *p* < 0.001). The majority of patients who died (415/468, 88.7%, 11 missing) had therapeutic limitations regarding ICU admission.

**Table 3:** Patient Outcomes during the two successive waves.

Outcomes	Overall (n = 2′983)	First wave (n = 1′014)	Second wave (n = 1′969)	*p* value
In-hospital mortality, n (%)	479 (16.1)	165 (16.3)	314 (16.0)	0.819
By age groups, n (%)				0.84*
< 65 years	32 (3.3)	14 (3.4)	18 (3.2)	0.88
65–85 years	203 (16.5)	69 (18.7)	134 (15.5)	0.164
> = 85 years	244 (31.9)	82 (36.30)	162 (30.1)	0.092
Discharge from hospital, n (%)				0.128
Death during hospital stay	396 (13.3)	137 (13.5)	259 (13.1)	
To home	609 (20.4)	227 (22.4)	382 (19.4)	
To post-acute care facility	1′978 (66.3)	650 (64.1)	1′328 (67.5)	
Mean total hospital stay in days (± SD, median, range)	37.6 (± 58.8, 15.1, 1–488)	40.9 (± 75.3, 13.0, 1.1–488)	35.8 (± 48.1, 16.1, 1.0–290)	0.002**
Patients admitted to ICU, n (%)	287 (9.6)	141 (13.9)	146 (7.4)	< 0.001
Patients admitted to IMCU, n (%)	749 (25.1)	226 (22.3)	523 (26.6)	0.011
Mean hospital ICU stay in days (± SD, median, range) (n = 287)	12.3 (± 11.2, 10, 0–80)	13.5 (± 9.6, 12, 0–46)	11.1 (± 12.6, 7: 0–80)	0.001**
Mean hospital IMCU stay in days (± SD, median, range) (n = 749)	4.3 (± 6.5, 2, 0–97)	4.3 (± 6.2, 2, 0–52)	4.3 (± 6.7, 2, 0–97)	0.953**
Pneumothorax during hospital stay, n (%) (n = 2′796)	21 (0.75)	5 (0.5)	16 (0.9)	0.364

*p value for interaction*Mann–Whitney test.

### In-hospital mortality

In univariate analysis, older age, male gender, the Charlson comorbidity index > 1, renal failure, increased C-reactive protein, SpO_2_/FiO_2_ ratio < 3.70 and increased respiratory rate were all associated with in-hospital mortality (Table 4). Corticosteroids were associated with an increased mortality in univariate analysis (HR 1.89; 95% 95% CI 1.58 to 2.26). In the multivariable analysis including 2119 patients, age, male gender, renal failure, SpO_2_/FiO_2_ ratio < 3.70 and increased respiratory rate remained independently associated with in-hospital mortality. A sensitivity analysis using continuous variables yielded similar findings (Supplementary Table 1). Hospitalization during the second wave was independently associated with a 25% relative risk reduction of death (HR 0.75; 95% CI 0.59 to 0.96) (Table 4). This reduction was further increased when corticosteroids were added to the model (HR 0.47; 95%CI 0.34 to 0.66). Corticosteroids remained independently associated with an increased in-hospital mortality in multivariable analysis (HR 1.96; 95%CI 1.40 to 2.76). Exploratory analyses showed in univariate analyses that patients receiving corticosteroids had significantly more comorbidities and had lower SpO_2_/FiO_2_ ratios (Supplementary Table 2). In a multivariable logistic regression model, the odds to be treated with corticosteroids were significantly increased with a higher number of comorbidities, the severity of hypoxemia (SpO_2_/FiO_2_ < 3.70 or between 3.70 and 4.50), abnormal CRP and high respiratory rate (Supplementary Table 3).

**Table 4:** Factors associated with in-hospital mortality during the two waves of COVID-19.

Variables	Univariate analyses*		Multivariable analyses*			
	Subhazard ratio (95%CI)	*p* value***	*Without steroids*		*With steroids*	
			Subhazard ratio (95%CI)	*p* value***	Subhazard ratio (95%CI)	*p* value***
First wave, (ref)	1.00		1.00		1.00	1
Second	0.96 (0.80–1.16)	0.675	0.75 (0.59–0.96)	0.020	0.47 (0.34–0.66)	<0.001
Age, years	1.06 (1.05–1.07)	< 0.001	1.08 (1.07–1.09)	< 0.001	1.08 (1.07–1.09)	< 0.001
Male Gender (ref. female)	1.73 (1.43–2.08)	< 0.001	1.62 (1.27–2.06)	< 0.001	1.60 (1.25–2.03)	< 0.001
Charlson comorbidity		< 0.001		0.080		0.116
Index		–		–		–
0 (ref.)	1.00	0.120	1.00	0.029	1.00	0.049
1–2	1.22 (0.95–1.58)	< 0.001	0.73 (0.55–0.97)	0.749	0.75 (0.56–0.99)	0.725
3–4	1.70 (1.32–2.21)	< 0.001	1.05 (0.78–1.42)	0.509	1.06 (0.78–1.42)	0.376
> 5	1.74 (1.31–2.30)		0.90 (0.65–1.24)		0.86 (0.62–1.20)	
Obesity	0.72 (0.30–1.73)	0.463	0.81 (0.28–2.35)	0.695	0.73 (0.25–2.14)	0.567
Creatinine ≥ 106 µmol/L (ref < 106)	2.68 (2.22–3.24)	< 0.001	1.30 (0.99–1.70)	0.062	1.30 (0.99–1.70)	0.056
Urea ≥ > 7.5 mmol/L (ref. < 7.5)	3.16 (2.61–3.82)	< 0.001	1.38 (1.04–1.82)	0.024	1.37 (1.04–1.81)	0.026
Categories of SpO_2_/FIO_2_		–		–		–
< 3.70 (ref)	1.00		1.00		1.00	
3.70–4.50	0.53 (0.42–0.67)	< 0.001	0.45 (0.34–0.60)	< 0.001	0.52 (0.39–0.70)	< 0.001
> 4.50	0.42 (0.32–0.54)	< 0.001	0.66 (0.47–0.93)	0.017	0.78 (0.55–1.10)	0.152
CRP ≥ 10 mg/L (ref. < 10)	2.06 (1.44–2.94)	< 0.001	1.56 (0.99–2.45)	0.054	1.42 (0.90–2.24)	0.130
Respiratory rate ≥ 20/min (ref. < 20)	1.78 (1.24–2.54)	0.002	1.62 (1.04–2.50)	0.032	1.45 (0.94–2.26)	0.096
Corticosteroids (ref. no)			–	–		
Yes	1.89 (1.58–2.26)	< 0.001			1.96 (1.40–2.76)	< 0.001

## Discussion

Two successive COVID-19 waves occurred in Geneva in spring and autumn 2020. Compared to the first wave, the second wave was characterized by an almost twice higher number of hospitalised patients. Patients tended to be older, more comorbid and had increased markers of disease severity. Nevertheless, adjusted in-hospital mortality rate was reduced by 25% and ICU admission by almost 50% during the second wave compared to first wave. Despite the increased difficulty to face an increased number of admissions, this finding probably illustrates the rapid adaptation of our hospital to an unprecedented pandemic, and the rapid improvement in the management of COVID-19 patients. This finding deserves several comments.

First, these differences might be explained by other factors than therapeutic management of COVID-19 patients such as changes in SARS-CoV-2 virulence or population immunity. However, the two successive waves occurred over several months and although some variants emerged in Europe at the beginning of the summer 2020, none were considered variants of interest, in the absence of evidence of increased transmissibility or virulence^18^. Moreover, SARS-CoV-2 seropositivity remained low after the first COVID-19 wave in the Geneva area, especially among elderlies^19^ and the first vaccination was administered on December 23rd 2020 in Switzerland: protection against severe disease conferred by pre-existing immunity was therefore unlikely to contribute to the between-waves differences.

As previously discussed, the main therapeutic changes between the two waves consisted in the choice of antiviral/anti-inflammatory therapies and respiratory support strategy. Hydroxychloroquine and lopinavir/ritonavir were used as therapeutic agents during the first months of the pandemic although these treatments appeared largely ineffective in subsequent studies^20–24^. However, they appear to have relatively favourable safety profile and are unlikely to explain the increased mortality observed during the first wave. Anti-IL6 treatments and remdesivir were used in very few patients, precluding any comparisons.

Corticosteroids were largely more prescribed (58 vs. 9%) during the second wave. The RECOVERY study, published in July 2020 reported a 17% relative rate reduction in mortality among patients hospitalized with COVID-19^6^. Similarly, Tomazini et al. reported a benefit in terms of increase of 2.6 ventilator-free days among patients with moderate to severe COVID-19 pneumonia who received corticosteroids in comparison to patients who received standard care alone^25^. The magnitude of treatment effect estimate in the RECOVERY trial was similar to the mortality reduction observed in our study. However, corticosteroids were not associated with a mortality reduction in our multivariable analysis. On the opposite, corticosteroids were associated with an increased HR of mortality in our univariate and multivariate model. Given the observational design of our study, this unexpected observation is suggestive of a selection bias or an inverse causality, due to the preferential administration of corticosteroids to the most severe patients. Indeed, as steroids were not routinely recommended for hospitalized patients during the first wave, they were selectively administered during this period to the more severe patients. Similarly, as steroids were routinely recommended during the second wave for patients requiring supplemental oxygen, the majority of patients not receiving steroids had preserved SPO_2_/FiO_2_ ratios. In our posthoc exploratory analysis, we found that patients receiving corticosteroids were significantly more likely to have several comorbidities and more severe hypoxemia.

Another potential explanation to the significant in-hospital mortality reduction in our study was the change regarding respiratory support strategies between the two waves. A striking result of our observation was the reduction by about one half of ICU admission and receipt of Intensive mechanical ventilation (IMV) during the second wave. Despite reluctance to provide non-invasive respiratory supports during the first weeks of the pandemic due to aerosolisation hazards, growing experience and evidence suggested that these therapies were safe when used in monitored units with adequate personal protection equipment and beneficial in terms of need for IMV and potentially mortality^8,9,26–28^. In a multicentric randomized controlled trial, Ospina et al. reported a 38% relative reduction of intubation hazard among COVID-19 patients treated with HFNO compared to conventional oxygen therapy^26^ and Grieco et al. suggested a possible further reduction of intubations among patients receiving Helmet NIV compared to HFNO.

While early intubation was the rule at the beginning of the pandemic at our institution, a delayed intubation strategy was progressively adopted in order to avoid IMV-associated complications and preserve our ICU capacity. According to this strategy, patients without therapeutic limitations regarding ICU or IMCU criteria were systematically admitted to the IMCU when requiring a FiO_2_ > 50%. Treatment in the IMCU included HFNO (60L/min, FiO_2_ titrated to maintain SpO_2_ > 90%), CPAP (8–12 cm/H2O 4 × 2 h/day) and awake prone position which was repeated according to patient tolerance and improvement in SpO_2_/FiO_2_. This strategy probably contributed to reduce the need for IMV and its associated complications. As a consequence, only the most severe patients were admitted to the ICU which is illustrated by an increased mortality in this subgroup during the second wave (33.6 vs. 25.5%).

The reported mortality in our cohort was lower than reported in other European countries, for example France (first wave 16.2%, second wave 17.7%) or Germany (first wave 19.1%, second wave 19.8%) which may results from difference in populations admitted to the hospital and systems of care^29,30^. Interestingly, no mortality reduction was observed across the two waves in these latter countries, whereas a large North American database reported an important reduction of critical care admission (30.9% to 13.3%) among hospitalized patients from spring to November 2020, but did not adjust for demographic variables, comorbidities or markers of disease severity^31^. Another cohort including 51 510 COVID patients reported a mortality reduction over time for patients with a positive rtPCR testing, but not in the group of clinically diagnosed COVID-19 infections^32^. A previous study using the COVID-19 Hospital based Surveillance (CH-SUR) database including 16 984 patients in Switzerland reported an adjusted mortality reduction of 30% between the two first COVID-19 waves in Switzerland (HR 0.70; 95%CI 0.63 to 0.78)^33^. However, this result was adjusted mainly on demographic variables and comorbidities but did not take into account markers of disease severity or administered treatments.

An important finding of our study was that the vast majority of deceased patients died without being admitted to the ICU. This illustrates the pivotal role of acute care and Geriatrics units in the selection of patients requiring ICU admission and the importance of the collaboration and dialogue with ICU physicians. Interestingly, the proportion of patients with therapeutic limitations regarding ICU admission increased during the second wave when the number of hospitalized patients was at its peak, constraining the Swiss medical Academy to update its triage rules in the perspective of a possible shortage of ICU resources. (https://sbv-fsa.ch/sites/default/files/2020-11/ASSM_criteres-triage_4-nov-2020.pdf). However, as the state of resource shortage was not officially declared during the second COVID-19 wave, decision regarding therapeutic limitations was left at the discretions of the treating physicians. The increased proportion of patients with therapeutic limitations during the second wave may therefore reflect implicit triage by physicians in charge and/or better identification of patients requiring ICU.

Our study has several limitations: First, the cross-sectional design did not allow to determine the respective contribution of the different aspects of patient management on patient outcomes. It is likely that the reduced mortality rate observed during the second wave was multifactorial and encompasses antiviral/anti-inflammatory treatments, respiratory support strategy and a global better knowledge of COVID-19 patients and identification of patients requiring more aggressive therapy. Second, our study was monocentric and conducted at a tertiary care hospital which limits the generalisability of our findings to other settings. However, we were able to adjust not only for demographics or co-morbidities but also for disease severity across the successive waves which was not performed in previous studies. Moreover, we included consecutive patients to limit potential selection bias. We therefore believe that this observation allows to evaluate the real-life impact of therapeutic adaptations on patient outcomes during the first waves of the pandemic and illustrates the rapid adaptation of health systems and immediate implementation of emerging evidence during an unprecedented public health challenge in the recent history. We believe that the multimodal strategy to disseminate and implement emerging evidence at our hospital may be of interest for other institutions as well as the pivotal role of intermediate care units to monitor patients with worsening hypoxemic respiratory failure and provide non-invasive respiratory support. These observational findings regarding the role of non-invasive respiratory support for acute hypoxemic respiratory failure confirm the reduction of the need for invasive ventilation reported by several RCTs which may be of importance in the context of potential future respiratory pandemics. Further research is warranted to establish the best respiratory strategy and time for intubation.

## Conclusion

Adaptation of therapeutic strategies and patient orientation including replacement of lopinavir/hydroxychloroquine by corticosteroids and higher admission to the IMCU to receive non-invasive respiratory support was associated with a reduction of the risk of in-hospital mortality in multivariable analysis, ICU admission and LOS during the second wave of COVID-19 despite an increased number of admitted patients. Non-invasive respiratory support and IMCUs may have a pivotal role to preserve ICU capacity in the global context of emerging COVID-19 variants or other respiratory pathogens that may lead to future respiratory pandemics.

## Supplementary Information

Supplementary Tables.

## Data Availability

The datasets generated during and/or analysed during the current study are available from the corresponding author on reasonable request.
