# Supplementary material for: Trends in management and outcomes of COVID patients admitted to a Swiss tertiary care hospital
Source: Sci Rep. 2023 Apr 12;13:6013. doi: 10.1038/s41598-023-32954-1 (PMC10096110; doi:10.1038/s41598-023-32954-1)
Supplement: Supplementary file 1 — Supplementary Tables. [file 41598_2023_32954_MOESM1_ESM.docx]

**Supplementary Table 1.** Factors associated with in-hospital mortality during the two waves of COVID-19 (continuous variables)

|  | **Univariate analyses^*^** | | **Multivariable analyses^*^** | | | |
| --- | --- | --- | --- | --- | --- | --- |
|  |  |  | ***Without steroids*** | | ***With steroids*** | |
| **Variables** | **Subhazard ratio (95%CI)** | **p-value^***^** | **Subhazard ratio (95%CI)** | **p-value^***^** | **Subhazard ratio (95%CI)** | **p-value^***^** |
| First Wave, (ref)  Second | 1.00  0.96 (0.80-1.16) | 0.675 | **0.76 (0.60-0.97)** | 0.028 | **0.52 (0.37-0.73)** | <0.001 |
| Age, years | 1.06 (1.05-1.07) | <0.001 | 1.09 (1.07-1.10) | <0.001 | 1.09 (1.07-1.10) | <0.001 |
| Male Gender (ref. female) | 1.73 (1.43-2.08) | <0.001 | 1.62 (1.27-2.08) | <0.001 | 1.60 (1.25-2.05) | <0.001 |
| Charlson comorbidity index | 1.08 (1.05-1.12) | <0.001 | 1.01 (0.97-1.06) | 0.609 | 1.00 (0.96-1.05) | 0.885 |
| Obesity | 0.72 (0.30-1.73) | 0.463 | 0.66 (0.22-1.96) | 0.450 | 0.65 (0.22-1.93) | 0.434 |
| Creatinine, µmol/L | 1.002 (1.001-1.003) | <0.001 | 1.00 (0.99-1.00) | 0.186 | 1.00 (0.99-1.00) | 0.168 |
| Urea, mmol/L | 1.06 (1.05-1.08) | <0.001 | 1.02 (0.99-1.04) | 0.082 | 1.02 (0.99-1.04) | 0.080 |
| SpO2/FIO2 ratio | 0.65 (0.60-0.71) | <0.001 | 0.68 (0.60-0.77) | <0.001 | 0.72 (0.63-0.82) | <0.001 |
| CRP, mg/L | 1.004 (1.003-1.005) | <0.001 | 1.002 (1.001-1.004) | 0.004 | 1.00 (1.00-1.00) | 0.006 |
| Respiratory rate, /min | 1.02 (1.01-1.03) | <0.001 | 1.01 (0.99-1.02) | 0.163 | 1.01 (0.99-1.02) | 0.249 |
| Corticosteroids (ref. no)  Yes | 1.89 (1.58-2.26) | <0.001 | - | - | **1.76 (1.25-2.48)** | 0.001 |

**Supplementary Table 2.** Comparison of patient characteristics and baseline measurements between treated and not treated by steroids, stratified on the wave of Covid-19 pandemic**.**

|  | **First wave** | | | **Second wave** | | |
| --- | --- | --- | --- | --- | --- | --- |
| **Variables** | **Steroids**  **(n=92, 9.1%)** | **No steroids**  **(n=921)** | **p-value** | **Steroids**  **(n=1144, 58.1%)** | **No steroids**  **(n=826)** | **p-value^***^** |
| Death during hospital stay, n (%) | 25 (27.2) | 140 (15.2) | 0.003^*^ | 244 (21.3) | 70 (8.5) | <0.001^*^ |
| Mean age (±SD, median, range), years | 71.6 (±14.3, 73: 61.5-81.5) | 68.0 (±18.5, 70: 55-83) | 0.076^**^ | 73.5 (±14.9, 76: 65-85) | 69.4 (±20.8, 77: 55-86) | <0.001^**^ |
| Sex, n (%)  Female  Male | 36 (39.1)  56 (60.9) | 431 (46.8)  490 (53.2) | 0.160^*^ | 450 (39.3)  694 (60.7) | 465 (56.3)  361 (43.7) | <0.001^*^ |
| Mean Charlson indice (±SD, median, range) (n=2’632) | 4.55 (±2.9, 4: 3-6) | 1.75 (±2.3, 1: 0-3) | <0.001^**^ | 2.15 (±2.4, 1: 0-4) | 2.06 (±2.4, 1: 0-3) | 0.5211^**^ |
| Charlson comorbidity index, n (%)  0  1-2  3-4  >=5 | 8 (9.4)  13 (15.3)  23 (27.1)  41 (48.2) | 359 (44.1)  201 (24.7)  155 (19.0)  99 (12.2) | <0.001^*^ | 364 (32.7)  346 (31.1)  241 (21.7)  161 (14.5) | 212 (34.1)  201 (32.4)  114 (18.4)  94 (15.1) | 0.442^*^ |
| Mean BMI (±SD, median, range) | 27.4 (±6.4, 27.0: 22.6-31.4) | 26.9 (±6.6, 26.3: 22.6-30.5) | 0.5227^**^ | 27.2 (±5.4, 26.5: 23.3-30.2) | 25.6 (±6.3, 24.7: 21.1-29.4) | <0.001^**^ |
| Categories of BMI, n (%)  <20  [20 – 25[  [25-30[  [30-40[  [40… | 6 (9.5)  19 (30.2)  17 (27.0)  18 (28.6)  3 (4.8) | 76 (13.7)  157 (28.3)  166 (29.9)  138 (24.9)  18 (3.2) | 0.797^*^ | 53 (6.8)  250 (32.0)  278 (35.6)  185 (23.7)  15 (1.9) | 118 (18.8)  210 (33.5)  160 (25.5)  122 (19.5)  17 (2.7) | <0.001^*^ |
| Abnormal creatinine (≥120 µmol/L for men, ≥92 for women) | 23 (27.1) | 182 (22.8) | 0.374^*^ | 339 (31.0) | 137 (24.2) | 0.003^*^ |
| Abnormal urea (≥8.5 mmol/L) | 25 (29.8) | 179 (22.2) | 0.119^*^ | 358 (33.3) | 143 (25.5) | 0.001^*^ |
| Categories of SpO2/FIO2, n (%)  <3.70  3.70-4.50  >=4.50 | 45 (58.4)  19 (24.7)  13 (16.9) | 382 (44.4)  241 (28.0)  237 (27.6) | 0.042^*^ | 727 (68.3)  250 (23.5)  88 (8.3) | 67 (8.8)  289 (37.7)  410 (53.3) | <0.001^*^ |
| CRP ≥ 10mg/L, n (%) | 482 (95.4) | 686 (86.6) | 0.020^*^ | 1007 (93.4) | 368 (69.7) | <0.001^*^ |
| Respiratory rate ≥20 n (%) | 87 (94.6) | 818 (88.8) | 0.088^*^ | 1086 (94.9) | 669 (81.0) | <0.001^*^ |
| Confusion, n (%) | 4 (4.4) | 17 (1.8) | 0.108^*^ | 53 (4.6) | 24 (2.9) | 0.051 |
| Mean 1^st^ hospital stay in days (±SD, median, range) | 25.8 (±20.5, 23.5: 9-36.5) | 13.5 (±22.4, 9: 5-16) | 0.003^**^ | 16.0 (±15.0, 12: 7-20) | 14.5 (±16.2, 10: 4-19) | <0.001^**^ |
| ICU admission, n (%) | 40 (43.5) | 101 (11.0) | <0.001^*^ | 121 (10.6) | 25 (3.0) | <0.001^*^ |
| IMCU admission, n (%) | 46 (50.0) | 180 (19.5) | <0.001^*^ | 411 (35.9) | 112 (13.6) | <0.001^*^ |
| Mean ICU stay in days (±SD, median, range) (n=287) | 19.8 (±8.6, 18.5: 13.5-27.5) | 11.0 (±8.8, 10: 6-15) | <0.001^**^ | 11.3 (±12.3, 7: 2-17) | 10.1 (±13.8, 4: 2-15) | 0.4395^**^ |
| Mean IMCU stay in days (±SD, median, range) | 8.7 (±9.8, 6: 2-12) | 3.2 (±4.3, 2: 1-4) | <0.001^**^ | 5.2 (±7.2, 4: 1-7) | 1.0 (±2.0, 0: 0-1) | <0.001^**^ |
| Mean time from admission to outcome or censoring, days (±SD, median, range) | 27.1 (±22.5, 23.9: 9.3-37.2) | 14.4 (±25.8, 9: 4.9-16.6) | <0.001^**^ | 17.4 (±20.7, 12.2: 7.5-20.0) | 15.6 (±19.4, 10.0: 4.2-20.1) | <0.001^**^ |

**Supplementary Table 3.** Association between patient characteristics or baseline measurements and treatment by corticosteroids (multivariable analysis).

| **Variables** | **Odds ratio** | **95% CI** | **p-value^*^** |
| --- | --- | --- | --- |
| Age, years | 0.99 | 0.99-1.00 | 0.386 |
| Gender  Female  Male | 1.00  1.16 | -  0.89-1.50 | 0.272 |
| **Charlson comorbidity index and wave (interaction)**  **First wave**  **0**  **1-2**  **3-4**  **>=5** | **1.00**  **2.46**  **4.78**  **14.75** | **-**  **0.91-6.66**  **1.87-12.25**  **6.09-35.77** | **<0.001**  **<0.001**  **-**  **0.077**  **0.001**  **<0.001** |
| **Second wave**  **0**  **1-2**  **3-4**  **>=5** | **1.00**  **0.66**  **1.01**  **0.84** | **-**  **0.47-0.95**  **0.67-1.51**  **0.54-1.31** | **0.099**  **-**  **0.024**  **0.969**  **0.444** |
| Obesity (Charlson index) | 1.55 | 0.61-3.96 | 0.357 |
| Abnormal creatinine (≥120µmol/L for men, ≥92 for women) | 1.00 | 0.70-1.43 | 0.997 |
| Urea ≥8.5 mmol/L | 1.04 | 0.72-1.50 | 0.843 |
| **Categories of SpO2/FIO2 and wave (interaction)**  **First wave**  **>=4.50**  **<3.70**  **3.70-4.50** | **1.00**  **0.93**  **1.05** | **-**  **0.43-2.01**  **0.44-2.53** | **<0.001**  **0.937**  **-**  **0.863**  **0.907** |
| **Second wave**  **>=4.50**  **<3.70**  **3.70-4.50** | **1.00**  **26.19**  **2.83** | **-**  **17.23-39.80**  **1.96-4.09** | **<0.001**  **-**  **<0.001**  **<0.001** |
| **CRP ≥**10mg/L | **2.43** | **1.66-3.57** | **<0.001** |
| **Respiratory rate ≥**20/min | **2.16** | **1.40-3.33** | **<0.001** |
| Confusion | 1.61 | 0.85-3.06 | 0.147 |
